# Evidence for a force favoring GC over AT at short intronic sites in *Drosophila simulans* and *Drosophila melanogaster*

**DOI:** 10.1093/g3journal/jkab240

**Published:** 2021-07-14

**Authors:** Ben Jackson, Brian Charlesworth

**Affiliations:** School of Biological Sciences, Institute of Evolutionary Biology, University of Edinburgh, Edinburgh EH9 3FL, UK

**Keywords:** evolution, GC-biased gene conversion, mutation, *Drosophila*

## Abstract

Population genetics studies often make use of a class of nucleotide site free from selective pressures, in order to make inferences about population size changes or natural selection at other sites. If such neutral sites can be identified, they offer the opportunity to avoid any confounding effects of selection. Here, we investigate evolution at putatively neutrally evolving short intronic sites in natural populations of *Drosophila melanogaster* and *Drosophila simulans*, in order to understand the properties of spontaneous mutations and the extent of GC-biased gene conversion in these species. Use of data on the genetics of natural populations is advantageous because it integrates information from large numbers of individuals over long timescales. In agreement with direct evidence from observations of spontaneous mutations in *Drosophila*, we find a bias in the spectrum of mutations toward AT basepairs. In addition, we find that this bias is stronger in the *D. melanogaster* lineage than in the *D. simulans* lineage. The evidence for GC-biased gene conversion in *Drosophila* has been equivocal. Here, we provide evidence for a weak force favoring GC in both species, which is correlated with the GC content of introns and is stronger in *D. simulans* than in *D. melanogaster*.

## Introduction

Population genetics studies often make use of a class of nucleotide site that is considered to be free from selective pressures, for the purpose of making inferences about the demographic history of a population (*e.g.*, Fagundes *et al.* 2007; Obbard *et al.* 2012; Garud *et al.* 2015). Such sites are also used as a neutral comparator for the purpose of estimating the parameters of natural selection acting on other types of sites, while controlling for other processes that affect the allelic composition of populations, such as population size changes, genetic drift, and mutation. For example, the McDonald-Kreitman test and its extensions (McDonald and Kreitman 1991; Fay *et al.* 2001; Smith and Eyre-Walker 2002; Welch 2006; Messer and Petrov 2013) rely on a class of sites that are assumed to evolve neutrally, whose relative levels of between-species divergence and within-species variability are contrasted to those for a putatively selected class, generating an estimate of the fraction of substitutions that have been fixed by positive selection as opposed to drift. More recent methods for inferring the distribution of fitness effects of new mutations also rely on a neutrally evolving class of sites, especially to correct for the effects of past population changes (Keightley and Eyre-Walker 2007; Boyko *et al.* 2008; Eyre-Walker and Keightley 2009; Schneider *et al.* 2011; Galtier 2016; Kim *et al.* 2017; Tataru *et al.* 2017).

In a number of species of *Drosophila*, there is evidence for the functional significance of a large fraction of the genome, including the action of both purifying and positive selection on intronic and intergenic sites (Andolfatto 2005; Haddrill *et al.* 2005; Halligan and Keightley 2006; Haddrill *et al.* 2008; de Procé *et al.* 2012; Vogl and Bergman 2015). Synonymous changes may also be subject to weak selection for preferred codons, which can affect allele frequencies in species with sufficiently large population sizes for such selection to be effective, including *Drosophila melanogaster* and *Drosophila* *simulans* (Begun 2001; Vicario *et al.* 2007; Jackson *et al.* 2017; Machado *et al.* 2020).

A candidate for a class of neutral nucleotide site in *Drosophila* are the 8–30 basepair regions that extend from the 5’ ends of introns shorter than 66 bp, but ≥23 bp after removing splice sites, which are referred to here as SI sites. These sites have been shown to have the highest between-species divergence and within-population diversity compared to other regions of the genome (Halligan and Keightley 2006; Parsch *et al.* 2010). These patterns are suggestive of a low level of purifying selection, and consequently a lack of functional importance. This makes them a good candidate for a neutral comparator of the type required by the methods mentioned above. For example, short introns have been used as a comparator for inferring strong purifying selection at fourfold degenerate sites in *D. melanogaster* (Lawrie *et al.* 2013; Machado *et al.* 2020), to fit demographic models to North American *D. melanogaster* in order to determine appropriate parameters for inferring selection from haplotype statistics (Garud *et al.* 2015), and to quantify population structure in European *D. melanogaster* (Kapun *et al.* 2020). Sites outside the central 8-30bp region but within short introns are probably more constrained because they are functionally important for mRNA splicing (Green 1986; Mount *et al.* 1992; Kennedy and Berget 1997; Halligan and Keightley 2006).

If SIs do indeed evolve in the absence of selective constraints, they provide an opportunity to investigate processes that affect the composition of genomes other than natural selection. Subsequent studies of evolution at SI sites in *Drosophila* have provided evidence for context-dependent mutational patterns (Clemente and Vogl 2012) and the possible action of GC-biased gene conversion (gBGC) (Vogl and Bergman 2015; Jackson *et al.* 2017). Evidence for gBGC in *Drosophila* genomes is equivocal, with some suggestion that it operates on the X chromosome in *D. simulans* (Haddrill and Charlesworth 2008) and *D. americana* (de Procé *et al.* 2012), and on both autosomes and the X chromosomes in *D. simulans* and *D. melanogaster* (Jackson *et al.* 2017), while other studies have found little or no evidence for it (Clemente and Vogl 2012; Comeron *et al.* 2012; Campos *et al.* 2013; Robinson *et al.* 2014).

Direct observations of spontaneous mutations in *D. melanogaster*, as well as analyses of rare segregating polymorphisms, show that mutation is biased toward GC to AT basepair substitutions (Assaf *et al.* 2017), and population genetic studies have suggested that the extent of this bias has increased at some point in the evolutionary past (Kern and Begun 2005; Zeng and Charlesworth 2010; Clemente and Vogl 2012). Laboratory studies of mutation are limited in power because mutations are rare—with a consensus mutation rate of approximately 5 × 10^−9^ per basepair (Assaf *et al.* 2017), we expect 0.7 mutations per haploid genome per generation in a genome containing 140 million base pairs. But an examination of the population genetics of natural populations provides the opportunity to integrate evidence from large numbers of individuals over long evolutionary timescales.

To investigate possible nonselective directional evolutionary processes in *Drosophila*, we have investigated evolution at autosomal short intron (SI) sites, using polymorphism data from populations from the putative ancestral ranges of *D. melanogaster* and *D. simulans*, as well as data on between-species divergence. Our study refines the analyses of Jackson *et al.* (2017), which primarily focussed on fourfold degenerate sites, since their analyses of SI sites were hampered by insufficient amounts of data and a poorer quality annotation of the *D. simulans* genome than the one used here. In addition, in contrast to Vogl and Bergman (2015) and Vogl and Mikula (2021), we analyze the relationship between the GC content of SIs and their evolutionary parameters and do not assume demographic equilibrium, using a larger set of *D. simulans* and *D. melanogaster* sequences than these previous studies. We show that a subset of SI sites are subject to directional evolutionary pressures, with GC alleles being favored over AT alleles at SI sites with the highest GC contents, suggesting the action of GC-biased gene conversion in both species. This has implications for the use of short introns as a neutrally evolving reference in population genetics, and also sheds light on the dynamics of genome evolution in *Drosophila*. The study also provides further evidence for the existence of a strong GC to AT mutational bias in Drosophila. Its magnitude appears to be independent of the GC content of short introns and has apparently increased along the *D. melanogaster* lineage following its divergence from the common ancestor of *D. melanogaster and D. simulans.*

## Materials and methods

### Sequence data from *D. simulans* and *D. melanogaster*

We have analyzed a previously published population sample of 21 lines of *Drosophila simulans*, derived from the putatively ancestral Madagascan population (the MD lines of Jackson *et al.* 2017). The sampling, maintenance, sequencing, and variant-calling procedures for these lines were fully described in Rogers *et al.* (2014), Jackson *et al.* (2017), and Becher *et al.* (2020). Briefly, publicly available raw read data in FASTQ format for these 21 lines were downloaded from the European Nucleotide Archive (study accession numbers: PRJEB7673 and PRJNA215932) and mapped to version 2.02 of the *D. simulans* reference genome (FlyBase release 2017_04) using BWA MEM (Li and Durbin 2009). We sorted, merged and marked duplicates on the resulting BAM files using Picard Tools version 2.8.3 (https://broadinstitute.github.io/picard/). Variants were called for each line individually using the HaplotypeCaller tool from GATK version 3.7 (McKenna *et al.* 2010) with the options –emitRefConfidence BP_RESOLUTION and –max-alternate-alleles 2. VCF files containing all 21 lines were generated from the output of HaplotypeCaller using the GATK tool GenotypeGVCFs. We treated sites that remained heterozygous within samples after inbreeding as follows: at each heterozygous site within a sample, one allele was chosen as the haploid genotype call at that site with a probability proportional to its coverage in the sample. The alternative allele was discarded (Jackson *et al.* 2017).

We also downloaded publicly available sequence data for 197 lines of *D. melanogaster* sampled from Zambia (ZI lines) from the Drosophila genome nexus (DGN) (https://www.johnpool.net/genomes.html) and converted these data to FASTA format using a custom shell script. Using the information reported in the supplement to Lack *et al.* (2015) we retained 69 ZI lines that showed no evidence of admixture with European populations. The sampling, sequencing and variant-calling procedures, and the procedure for defining admixture tracts for these ZI lines were described fully in Pool *et al.* (2012) and Lack *et al.* (2015). The ZI sample is maximally diverse and minimally affected by cosmopolitan admixture among the populations in the DGN (Lack *et al.* 2015), and also provides the largest sample of African *D. melanogaster* genomes.

### Between-species alignments

We used the multispecies alignment between *D. melanogaster*, *D. simulans* and *D. yakuba* from Zeng *et al.* (2019). Briefly, a multispecies alignment was performed between the reference genomes of *D. simulans* (v2.02), *D. melanogaster* (v5.57), and *D. yakuba* (v1.05) using the MULTI-Z pipeline described by Barton and Zeng (2018). Reference genomes were downloaded from FlyBase, and repeat regions were soft-masked using RepeatMasker (http://www.repeatmasker.org/) with the default database for *Drosophila*. Pairwise alignments were generated between *D. melanogaster* and *D. simulans*, and between *D. melanogaster* and *D. yakuba*, using LASTZ (Harris 2007), which were chained and netted using axtChain and chainNet (Kent *et al.* 2003). Single coverage was generated using single_cov2.v11 from the MULTIZ package (Blanchette *et al.* 2004) and the pairwise alignments were aligned with MULTIZ to create three-way multiple alignments.

### Defining short intronic sites

To define short intronic (SI) sites, we first carried out the following procedure separately for each of *D. melanogaster* and *D. simulans*. We used the information in the header lines of the FlyBase FASTA file of introns for version 2.02 (5.57) of the *D. simulans* (*D. melanogaster*) reference genome to extract coordinates of the 8–30 bp region of introns that were ≤ 65 bp in length, after checking that this region did not overlap with an exon, an intron of length more than 65 bp, or the non-8–30 bp portion of an intron of length ≤65 bp, using information from the gff format annotation of the *D. simulans* (*D. melanogaster*) reference genome version 2.02 (5.57) downloaded from FlyBase.

Using the resulting SI positions in each species and the whole genome alignment described above, we defined a set of homologous sites that were annotated as SI sites in both *D. simulans* and *D. melanogaster*, using the script non_ref_intersect.py from the WGAbed package (https://henryjuho.github.io/WGAbed/) and the bedtools subroutine intersectBed (Quinlan and Hall 2010), as well as additional custom shell and Python scripts. We generated an alignment for each *D. melanogaster* short intron region that overlapped with a *D. simulans* short intron region, yielding polymorphism data for the ZI and MD lines, as well as the corresponding sequences from each of the *D. melanogaster* v5.57, *D. simulans* v2.02 and *D. yakuba* v1.3 reference sequences.

At this stage, we retained SI sites only if the following additional conditions were met: they were located on an autosome in both *D. melanogaster* and *D. simulans*; there were no missing alleles in any of the three reference sequences; they were not soft-masked as repetitive in any of the three reference sequences; they did not overlap with an indel in the *D. simulans* variant callset; QUAL ≥30 in the *D. simulans* variant callset; they did not lie in a noncrossover region in either the *D. melanogaster* genome (as defined in Campos *et al.* 2012) or in the *D. simulans* genome (as defined in Becher *et al.* 2020). There were two reasons for the last procedure. First, the evolutionary processes in noncrossover regions are unusual, because of strong hitchhiking effects (Becher *et al.* 2020). Second, levels of polymorphism in these regions are very low, so that little information is provided by them. In total, we retained 167,147 autosomal SI sites for divergence-based analyses. For polymorphism-based analyses, such as those using derived allele frequencies or site frequency spectra (see below), we further excluded sites with any missing polymorphism data in the population under consideration. We retained 163,998 and 145,747 sites for polymorphism-based analyses of the MD lines and the 69 ZI lines, respectively. We used only data from autosomes, as there were too few X-linked SNPs to allow partitioning into different bins.

Our final dataset consisted of 9327 introns. For the purposes of comparing regions with different GC contents, we ordered the introns by increasing GC content and then divided them into five bins, so that each bin contained an approximately equal number of introns (1865 for the first four bins and 1867 for the final bin). We first obtained the GC content in the relevant species’ reference sequence at the coordinates that corresponded to the full 8–30 bp region of each short intron under consideration. Then we carried out the binning procedure above in two different ways. First, we took the mean of the GC content values for each homologous pair of introns and applied this single value when grouping both species’ SIs. This results in the same set of homologous sites in each bin for analyses of divergence and polymorphism in both lineages. Second, we grouped introns into species-specific bins, for *D. melanogaster* by using the GC content calculated from the *D. melanogaster* reference sequence, and for *D. simulans* by using the GC content calculated from the *D. simulans* reference sequence. This means that homologous sites may be assigned to different bins in analyses of the *D. simulans* lineage from those in analyses of the *D. melanogaster* lineage, but ensures a perfect relationship between within-species GC content and bin. Below, we refer to these two binning strategies as “mean” and “species,” respectively. The two GC contents are closely correlated, as is expected given the slow evolution of GC content (Supplementary Figure S1). In the Discussion, we argue that the method of binning by mean GC content is preferable to the species-specific method when analyzing substitution patterns; and vice versa for patterns of polymorphism. For completeness, we show the results for the opposite binning methods in the Supplementary material.

To obtain confidence intervals (CIs) around point estimates of statistics, we bootstrapped by sampling introns with replacement 1000 times until the bootstrapped sample was the same size as the observed sample. For each bootstrap sample, we recalculated the statistic of interest. We used the 2.5% and 97.5% quantiles of the resulting distribution as the upper and lower bounds of the 95% CI (Efron 1979).

### Analyses of between-species divergence

We used the GTR-NHb model of base substitution modified to generate sub-optimal ancestral sequences, as implemented in the baseml program of PAML version 4.8 (Yang 2007), in order to reconstruct the base content of the *melanogaster*-*simulans* (*ms*) ancestor, and counted substitutions along lineages according to the Expected Markov Counting method of Matsumoto *et al.* (2015). This method should be more accurate than maximum parsimony or use of a single best reconstruction under complex patterns of base substitution, which are likely apply to *Drosophila* (Matsumoto *et al.* 2015). We checked that the GTR-NHb fitted the data better than the stationary GTR model, also implemented in PAML, using likelihood ratio tests—this was true in all cases. For each bin, we ran baseml ten times and manually checked for convergence by examining the likelihood output of the model. In the results presented below, we refer to G and C alleles as strong (*S*) and to A and T alleles as weak (*W*). We categorized the number of substitutions from the *ms* ancestor to the extant *simulans* sequence or to the extant *melanogaster* sequence into the following classes: the total number of substitutions from strong to weak alleles, *N_S_*_>_*_W;_* the total number of substitutions from weak to strong alleles, *N_W_*_>_*_S;_* and the total number of substitutions from strong to strong alleles or from weak to weak alleles, *N_neu_*. We denote the number of GC sites in the ancestral sequence by *L_GC_* and the number of AT sites in the ancestral sequence by *L_AT_*. We define the substitution rate from strong to weak alleles as *r_S_*_>__*W*_ = *N_S_*_>__*W*_/*L_GC_*, and the substitution rate from weak to strong alleles as *r_W_*_>__*S*_ = *N_W_*_>__*S*_/*L_AT_*. We obtained the expected numbers of substitutions and the predicted ancestral base content by parsing the output of PAML, using custom scripts in R (R Core Team 2018).

### Analyses of polymorphism data

We divided SI sites into the same sets of five bins as used for the divergence-based analyses. For each population, we excluded sites with missing data in the polymorphism sample as well as segregating sites of more than two alleles, and then used est-sfs v2.03 (Keightley and Jackson 2018) to calculate the probability of the major allele being ancestral for each segregating site. We used the Kimura 2-parameter model of base substitution, which was found by Keightley and Jackson (2018) to perform just as well in *Drosophila* as a more complex 6-parameter model, and two outgroups (*D. yakuba* and one of either *D. melanogaster* or *D. simulans*, depending on the species to which the polymorphism data belonged) to run est-sfs. We carried out 10 maximum likelihood searches for each bin to check for convergence. Using the results, we constructed separate unfolded site-frequency spectra (SFSs) for segregating *S* >*W*, *W* >*S*, and neutral (*W* >*W* or *S *>* S*) mutations. We used these SFSs to calculate the mean derived allele frequency (DAF) for each class of change, and as an input for the method of Glémin *et al.* (2015) for estimating the mutation and selection parameters.

This method uses the three unfolded SFSs for segregating sites described above to estimate *γ* and *κ*, where *γ*  =  4 *N_e_s* is the scaled strength of selection for GC (*S*) alleles, and *κ* is the mutational bias parameter *u*/*v*. Here, *s* is the selection coefficient against heterozygotes for W and S alleles (semi-dominance is assumed), *u* is the mutation rate from *S* to *W*, and *v* is the mutation rate from *W* to *S*. The method is capable of taking into account polarization errors, which can lead to upwardly biased estimates of *γ* (Hernandez *et al.* 2007), by incorporating them into the model and estimating them jointly with the parameters of interest. It also corrects for demographic effects, by introducing nuisance parameters to adjust for distortions in the SFS due to demography (following Eyre-Walker *et al.* 2006). We estimated *γ* and *κ* using the R code provided in the supplement of Glémin *et al.* (2015). We refer to the models using this method with the same notation as in Glémin *et al.* (2015). These are model M0, with *γ*  =  0, and no correction for polarization errors; M1, with *γ *≠ 0 and no correction for polarization errors; and M0* and M1*, which are the equivalent models including a correction for polarization errors. We compared the different models using likelihood ratio tests.

### Computational methods

This work made use of GNU parallel (Tange 2011).

## Results

### Summary of polymorphism and divergence results

The population of *D. simulans* from its putatively ancestral range in Madagascar is more diverse than either population of *D. melanogaster—*mean nucleotide site diversity (*π*) at SI sites is 0.037 for the MD sample and 0.016 for the ZI sample (Table 1), which is in agreement with previous analyses (Jackson *et al.* 2017). The site frequency spectrum is more skewed toward rare variants in *D. simulans* than in *D. melanogaster*, as shown by the larger absolute value of Tajima’s *D* and proportion of singletons in the MD sample, compared to ZI (Table 1). The ratios of the proportions of singletons to their expected values under neutrality are 1.83 and 1.48 for MD and ZI, respectively. Consistent with this finding, recent population growth has been inferred for both of these populations (Zeng *et al.* 2019; Johri *et al.* 2020). This means that the assumption of equilibrium made by Vogl and Bergman (2015) and Vogl and Mikula (2021) in their analyses of a smaller *D. simulans* dataset could lead to errors in inference. Our use of the unfolded site frequency spectrum allowed the use of the inference procedure of Glémin *et al.* (2015), which contains an adjustment for demographic effects.

**Table 1:** Polymorphism statistics for SI sites (means with their 95% CIs)

Population	Nucleotide diversity (*π*)	Watterson’s *θ*	Tajima’s *D*	Proportion of singletons
MD	0.0371 (0.0365, 0.0377)	0.0513 (0.0507, 0.0521)	−1.16 (−1.133, −1.183)	0.513 (0.506, 0.519)
ZI	0.0164 (0.0159, 0.0168)	0.0195 (0.0191, 0.0199)	−0.566 (−0.517, −0.616)	0.308 (0.301, 0.317)

With all SI sites concatenated, the net divergence between *D. melanogaster* and *D. simulans* is 0.124 (0.121–0.126), 0.0655 (0.0637–0.0674) for the *D. melanogaster* branch, and 0.0582 (0.0567, 0.0598) for the *D. simulans* branch (the brackets indicate the CIs for the means). These values are nearly identical to the results of Parsch *et al.* (2010), who reported the divergence between *D. melanogaster* and *D. simulans* at the 8–30 bp region of introns <66 bp long to be 0.123, with the divergence along the *D. melanogaster* lineage equal to 0.064.

### Testing for fixation bias

**Figure 1:**
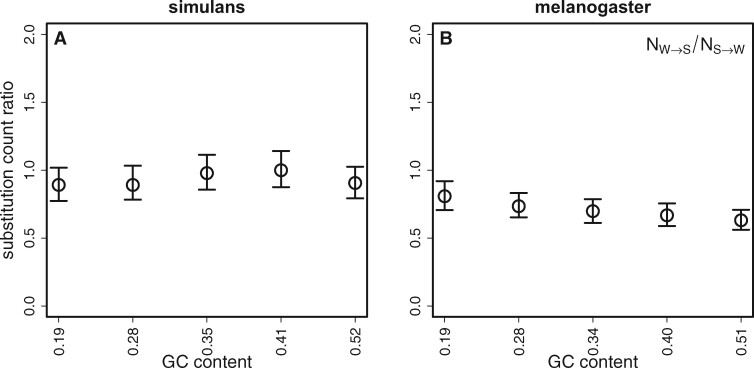
The ratio of substitution counts and its relationship with the GC content of short introns. A substitution count ratio of *N_W_*_>__*S*_/*N_S_*_>__*W*_ = 1 implies equilibrium base composition. Ratios were calculated for the *D. simulans* lineage (A) and the *D. melanogaster* lineage (B). Short intron sites were binned by the mean GC content of homologous introns in *D. melanogaster* and *D. simulans*. Error bars represent 95% CIs from 1000 bootstraps of the data in each bin.

If base composition is at statistical equilibrium under mutation, drift, and selection over the period of time covered by an evolutionary lineage, there should be equal numbers of substitutions from *S* (G or C) to *W* (A or T) alleles and from *W* to *S* alleles (Bulmer 1991; Akashi 1995; Charlesworth and Charlesworth 2010, p. 272). The results here suggest an equilibrium base composition in *D. simulans* autosomal SIs when we concatenate all SI sites. *N_W_*_>__*S*_ for all SI sites combined in *D. simulans* was 3500, and *N_S_*_>__*W*_ was 3584, which do not differ significantly from a ratio of 1:1 (χ^2^^* *^= ^* *^0.99, *df* = 1, *P *=* *0.32). Using the same methodology, we previously reported a slight overall AT-bias in substitutions in *D. simulans* autosomal short introns (χ^2^^* *^=^* *^5.55, *df* = 1, *P *=* *0.019) (Jackson *et al.* 2017). After binning SIs by their mean GC content across species, there was no obvious relationship between GC content and the ratio *N_W_*_>__*S*_/*N_S_*_>__*W*_. In *D. simulans*, the 95% CIs obtained by bootstrapping overlap unity for all bins (Figure 1A). This suggests that aggregating sites with different GC contents do not mask any substitution patterns that are specific to sequence context.

*D. melanogaster* shows an overall bias toward AT-fixation for all sites combined (*N_W_*_>__*S*_ = 3478, *N_S_*_>__*W*_ = 4760, χ^2^^* *^= 199, *df* = 1, *P *<* *0.001), which is in agreement with previous results (Akashi *et al.* 2006; Jackson *et al.* 2017). When *D. melanogaster* SI sites are binned by mean GC content, all of the bins exhibit an AT fixation-bias, which increases with increasing GC content (Figure 1B). This implies that *D. melanogaster* has experienced a change in the forces acting on GC content, such that its GC content is currently not at equilibrium; this could either be a change in mutational bias toward GC>AT mutations, or a reduction in the scaled intensity of a selective force or biased gene conversion favoring GC, possibly reflecting a reduction in effective population size. In contrast, *D. simulans* appears to be approximately in equilibrium. The comparable results for the species bins are shown in Supplementary Figure S2.

### Analyses of polymorphism data

The polymorphism data were used to investigate the parameters of mutation and selection acting on GC versus AT variants. In this case, binning SIs by the species-specific GC content is more appropriate than using their mean values across species, since it provides a better reflection of the sequence composition over the comparatively short time-scale experienced by currently segregating variants. The comparable results for the mean bins are shown in Supplementary Figure S3.

We examined the nature of the forces acting on polymorphic variants in two ways. First, for each bin, we calculated the mean derived variant frequency for different classes of mutations at segregating sites. These classes involved either GC to AT variants (*DAF_S_*_>__*W*_), AT to GC (*DAF_W_*_>__*S*_), or GC to CG or AT to TA (*DAF_neu_*). These statistics should shed light on the processes of interest in *Drosophila* genome evolution as follows. If the mutational process has shifted toward a greater GG>AT bias within the last 4 *N_e_* generations (the time frame relevant to polymorphism data), as has previously been suggested for *D. melanogaster* (Akashi *et al.* 2006; Jackson *et al.* 2017), we expect *DAF_S_*_>__*W*_ < *DAF_W_*_>__*S*_, because mutations from GC to AT should be younger on average, even under neutrality. Furthermore, if such a change in mutational bias were genome wide, we do not expect a relationship between DAF and GC content. In contrast, if gBGC or a selective force favoring GC is in operation, we expect to have *DAF_W_*_>__*S*_ > *DAF_neu_* > *DAF_S>W_* (Jackson *et al.* 2017). In addition, if the strength of such a force varies across the genome and has influenced its GC content, we expect this relationship to be stronger in regions of higher GC content—that is, *DAF_S_*_>__*W*_ should be negatively related to GC content and *DAF_W_*_>__*S*_ should be positively related to GC content.

**Figure 2:**
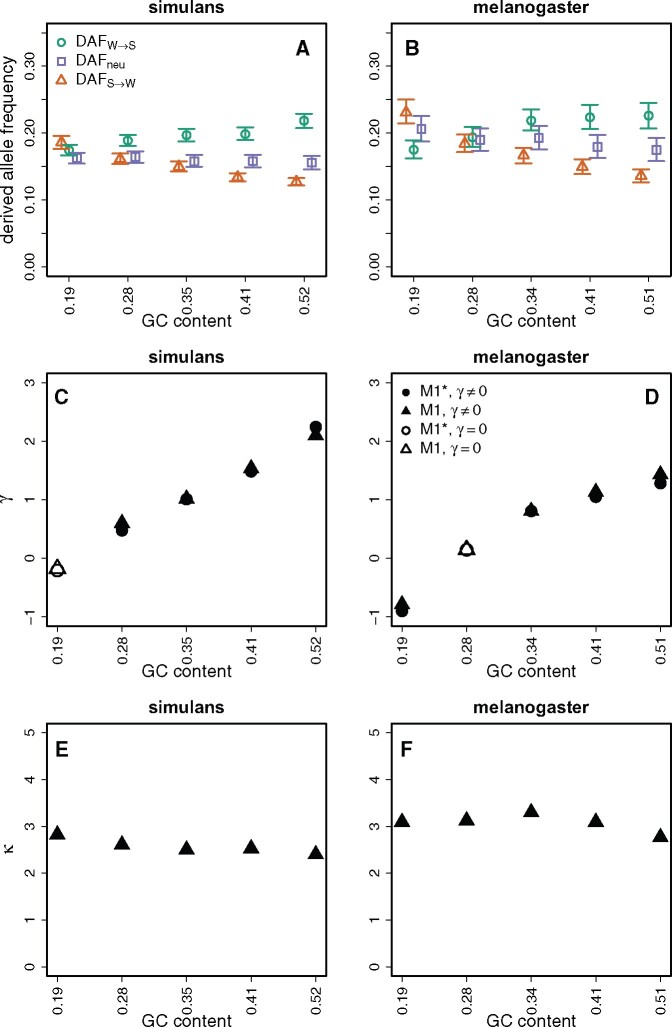
Analyses of polymorphism data and their relationship with GC content. Top row: Derived allele frequencies (DAF) for different types of mutations. DAF was calculated for the *D. simulans* MD population (A) and the *D. melanogaster* ZI population (B). The key for these two panels is shown in panel A. Middle row: Estimates of the strength of selection in favor of GC alleles ($\gamma =4N_{e}s$). Open symbols indicate values which are not significantly different from zero. Circular symbols denote models incorporating parameters that correct for polarisation error. Triangular symbols denote models not incorporating parameters that correct for polarisation error. *γ* was calculated for *D. simulans* (C) and *D. melanogaster* (D). The key for these two panels is shown in D. Bottom row: The estimates of the mutational bias parameter, *κ*, calculated using the method of Glémin *et al.* (2015). *κ* was estimated from the model M1 (polarisation errors not corrected for) with *γ*  ≠  0, for both *D. simulans* (E) and the *D. melanogaster* (F). Sites were binned by species-specific GC content. Error bars represent 95% CIs from 1000 bootstraps of the data in each bin.

This second pattern, suggestive of a force favoring GC, is indeed what we observe in *D. simulans* (Figure 2A). For the Zambian sample of *D. melanogaster*, we observe the pattern supporting a GC-favoring force in the top three highest GC content bins (Figure 2B).

Overall, these results suggest the presence of a GC-favoring force in *D. simulans* and, probably to a lesser extent, in *D. melanogaster*. In order to quantify this force, we used the method of Glémin *et al.* (2015) to calculate *γ *= 4 *N_e_s*, the scaled strength of selection or biased gene conversion favoring GC alleles. In no cases did models correcting for polarisation errors fit the data better than the equivalent model without corrections. This may be because the method we used to polarise segregating sites is less prone to mis-inference than methods such as maximum parsimony (Keightley and Jackson 2018). Both sets of models returned very similar values of *γ* within each species (Figure 2, C and D). Below, we report the results from models M0 and M1, which do not correct for polarization error (see *Materials and methods*).

In *D. simulans*, there is little evidence for a force favoring GC alleles in the lowest GC content bin. In the remaining four bins, the relationship between GC content and gamma is somewhat more pronounced, with *γ*  =  0.60 in the second-lowest GC bin, rising to *γ*  =  2.10 in the highest GC content bin (Figure 2C). For *D. melanogaster*, the lowest bin shows evidence for a force favoring AT, with *γ* = – 0.79 (Figure 2D). The second-lowest GC bin shows no evidence that either strong or weak alleles are preferred, and the top three bins show evidence for a force favoring GC. As was found for *D. simulans*, the relationship between GC content and *γ* is more pronounced for the species bins than the mean bins. Similar to the interpretations discussed for the DAF patterns, it seems likely that the action of gBGC or selection has diverged somewhat between the two lineages.

This method also allows the estimation of κ, the mutational bias parameter (Figure 2, E and F), which is estimated jointly with γ (Glémin *et al.* 2015). We report values of κ from model M1. In *D. melanogaster*, κ seems fairly insensitive to GC content. From the species bins, the values of κ are 3.09, 3.13, 3.30, 3.10, and 2.77 (Figure 2F). These values are close to the estimate derived from a meta-analysis of direct observations of spontaneous mutations in *D. melanogaster* mutation accumulation experiments, which was 3.35 (95% CIs: 3.00–3.76) (Assaf *et al.* 2017). In *D. simulans*, κ is somewhat lower, and seems to be slightly negatively correlated with GC content, with estimates 2.82, 2.61, 2.50, 2.53, and 2.40 (Figure 2E). These species differences are in agreement with the hypothesis of an increase in the GC to AT mutational bias in the *D. melanogaster* lineage relative to the *D. simulans* lineage, which has been proposed before (Takano-Shimizu 2001; Kern and Begun 2005; Zeng and Charlesworth 2010; Clemente and Vogl 2012).

### Patterns of substitution and their relationship with GC content

If a fraction of SI sites is subject to a weak force favoring GC over AT, this should be reflected in the patterns of substitution rates. As a null hypothesis, we might expect sites in the lowest GC content bins, where there is little evidence for a force favoring GC, to exhibit substitution rates that reflect the mutational bias inferred from polymorphism data and mutation accumulation experiments—under neutrality, substitution rates, and mutation rates are equal (Wright 1938; Kimura 1968). For higher GC content bins, where there is evidence for an advantage to GC, we expect a higher rate of substitution of GC alleles relative to the lower GC content bins. To investigate this, we reconstructed ancestral states using PAML (see *Materials and* *methods* for details), and counted the numbers of *S *>* W* and *W *>* S* substitutions along each lineage, in order to estimate the ratio of the two substitution rates, *R *=* r _S_*_>__*W*_/*r _W_*_>__*S*_, for both the *D. melanogaster* and *D. simulans* lineages. Note that *R*  =  *κ* under strict neutrality. As before, we used the mean bins for this analysis of substitution patterns; the results for the species bins are shown in Supplementary Figure S4.

**Figure 3:**
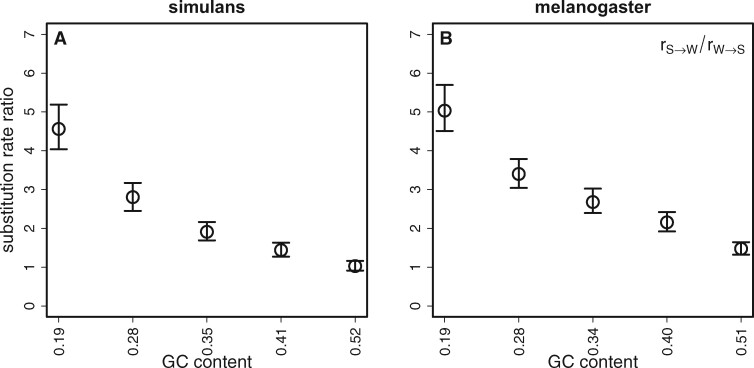
The ratio of substitution rates (*R = r_S>W_/r_W>S_*) and its relationship with GC content.R was calculated for the *D. simulans* lineage (A) and the *D. melanogaster* lineage (B). Sites were binned by the mean GC content of homologous introns in *D. melanogaster* and *D. simulans*. Error bars represent 95% CIs from 1000 bootstraps of the data in each bin.

In agreement with this hypothesis, *R* and GC content are negatively correlated in both *D. simulans* and *D. melanogaster* (Figure 3). However, the values of R for the lowest mean GC content bins are much higher than the values ofκ described above. For the lowest mean bins, *R* is equal to 4.56 and 5.03 in *D. simulans* and *D. melanogaster*, respectively (Figure 3, A and B). The absolute rates *r _S_*_>__*W*_ and *r _W_*_>__*S*_ are plotted in Supplementary Figure S5. The fact that *R *≫ *κ* for the mean bins with low GC content clearly requires an explanation, given the above evidence that these bins are evolving close to neutrally. One possibility is that there has been selection in these bins in favor of AT rather than GC along both lineages, consistent with the significantly negative value of *γ* for the lowest GC content species bin in *D. melanogaster* (Figure 2D). This would also explain why both the present and reconstructed ancestral GC contents of the low GC bins are much lower than the equilibrium GC content predicted on the basis of neutral evolution and the estimated *κ* value of approximately 3 (Figure 2); this is equal to 1/[1 + *κ exp(–γ*)], which takes a value of 0.25 with *γ *= 0 (Li 1987; Bulmer 1991; Charlesworth and Charlesworth 2010, p. 275).

Supplementary Table S1 shows that, for the lowest mean bin, the GC content for *D. simulans* is 0.195 and the GC content for *D. melanogaster* is 0.192. The GC contents of the ancestral sequence reconstructed by PAML are 0.197 for the lowest *D. simulans* and *D. melanogaster* mean bins. For equilibrium, such a low GC content requires *κ*  =  4.13 under neutrality, but a smaller value if there were some selection against GC, *e.g.*, with *κ*  =  2.8 (the species value for *D. simulans*), an equilibrium GC content of 0.20 requires *γ* = – 0.35. From Equation 2 of Jackson *et al.* (2017), this implies an *R-*value of 3.97, which is close to the value estimated for the *D. simulans* lineage. Alternatively, there could have been a shift downwards from an ancestral *κ* value of approximately 4 in both lineages, which would not be detected if it occurred prior to the period of time for which the polymorphism data are relevant (these only provide an estimate of the value of *κ* over the past 4 *N_e_* generations).

Another explanation is that the discrepancy between *R* and *κ* for the low GC bins is an artifact due to alignment errors in AT-rich regions, which might contain more repeat sequences (Bachtrog *et al.* 1999) and thus be harder to align. The *D. melanogaster* reference sequence is moderately more AT-rich in regions that we soft-masked for repeat content compared to the remainder of the genome (GC content for the masked regions = 39%, GC content for the remainder of the genome = 43%). However, the lowest GC content bins do not exhibit lower alignment scores according to the whole genome alignment MULTI-Z output, which suggests that there are no specific alignment problems for these regions (Supplementary Figure S6). As noted in the methods, we excluded sites masked as repetitive in either species from all of our analyses.

### No effect of polymorphism on patterns of divergence

Because the nucleotide diversity within these species represents an appreciable fraction of divergence between them (Table 1), we repeated our analyses of divergence after removing sites that were polymorphic in either the MD sample or the ZI sample, to approximate a dataset consisting only of fixed differences between *D. simulans* and *D. melanogaster*. Removing polymorphic sites had the effect of considerably reducing the substitution rates for both species, more so for *D. simulans* (compare Supplementary Figures S5 and S7). It had no effect on the patterns of *R* or the substitution count ratio (Supplementary Figure S7).

## Discussion

Understanding whether genome evolution involves gBGC or a selection pressure acting on the GC content of putatively functionless sequences is important for two reasons. First, it is needed for a complete understanding of the processes affecting the genetic composition of natural populations. Second, we expect forces of this nature to affect sites that are often used as comparators for detecting other evolutionary processes, such as selection on functionally important sites, changes in population size, and mutation. To date, the evidence for a GC-favoring force in *Drosophila* has been ambiguous. It has been claimed to be acting on the X chromosome of *D. simulans* (Haddrill and Charlesworth 2008) and *D. americana* (de Procé *et al.* 2012), and on both X chromosomes and autosomes in *D. simulans* and *D. melanogaster* (Jackson *et al.* 2017), while several other studies have failed to find support for it (Clemente and Vogl 2012; Comeron *et al.* 2012; Campos *et al.* 2013; Robinson *et al.* 2014). We have extended our previous work on this topic (Jackson *et al.* 2017) by focussing exclusively on SI sites, and by using a larger polymorphism sample in *D. melanogaster* than before, together with more complete annotation of the *D. simulans* genome. This allowed us to take the intersection of sites annotated as short introns in both species, which in turn allowed a direct comparison of the processes acting at homologous sites.

Overall, the analyses presented above suggest the existence of a GC-favoring force in both *D. simulans* and *D. melanogaster*, whose strength is positively related to the GC content of an intron, and which is on average stronger in *D. simulans*. This makes sense in the context of GC-biased gene conversion (gBGC), which is a recombination-association process whose evolutionarily effective strength is proportional to the product of the rate of change of allele frequency by gene conversion and the effective population size (*N_e_*) (Nagylaki 1983; Charlesworth and Charlesworth 2010, p. 529). On the basis of pairwise diversity at SI sites, *N_e_* is substantially higher for the *D. simulans* population compared to the *D. melanogaster* population, assuming that mutation rates are similar for the two species (Table 1 and Supplementary Figure S6), so that the evolutionarily effective strength of any deterministic force over the recent past should be larger in *D. simulans*, other things being equal. There are only weak relationships between SI site diversity and GC content (Supplementary Figure S6), with an observed ratio of π for the highest versus the lowest species bins of 0.79 for *D. simulans* and 0.84 for *D. melanogaster*. If mutation-selection-drift equilibrium is assumed, and the estimates of *κ* and *γ* for these species bins are inserted into Equation 15 of McVean and Charlesworth (1999), the predicted ratios are 0.93 and 1.47 for *D. simulans* and *D. melanogaster*, respectively. The agreement between the observed and predicted values is reasonably good for *D. simulans*, but there is a large discrepancy for *D. melanogaster*, possibly reflecting a larger departure from base composition equilibrium than in the case of *D. simulans*.

Nevertheless, the presence of a force favoring GC is suggested both by the analyses of polymorphism data, using estimates of the derived allele frequencies of different sorts of mutation and the site frequency spectrum based estimate of *γ* (Figure 2), as well as by the analyses of substitution rates (Figure 3). There is also evidence for a strong mutational bias in favor of GC>AT mutations, consistent with direct evidence from mutation accumulation data (Assaf *et al.* 2017). This bias is larger in *D. melanogaster* than *D. simulans*, and the evidence that base composition is close to statistical equilibrium in the latter but not the former suggests that there may have been a shift toward a stronger mutational bias in the *D. melanogaster* lineage, as has previously been suggested on the basis of somewhat weaker evidence (Kern and Begun 2005; Zeng and Charlesworth 2010; Clemente and Vogl 2012; Jackson *et al.* 2017). The mechanistic basis and evolutionary significance of such a shift are both unclear.

A potential cause of the association between GC content of SIs and *γ* is that there is a higher rate of biased gene conversion in genomic regions with higher rates of crossing over, leading to higher GC contents in such regions. In addition, the lower efficacy of selection with a lower rate of recombination, due to increased Hill-Robertson interference effects (*e.g.*, Charlesworth *et al.* 2010), might cause a lower value of *γ* in genomic regions with lower rates of crossing over. However, these explanations are inconsistent with the lack of evidence for an association between the GC content of introns or synonymous sites and the rate of crossing over in *D. melanogaster*, if noncrossover regions of the genome are excluded (Haddrill *et al.* 2007; Campos *et al.* 2012) (Supplementary Figure S8).

Our results show that there has been some divergence in GC content at a subset of the SI sites that are shared between *D. simulans* and *D. melanogaster*, with *D. simulans* SIs having slightly higher GC contents than the same introns in *D. melanogaster* (see the rows labeled “Mean” in Supplementary Table S1). Binning short introns by the mean GC content of homologous sites potentially has the effect of masking some of these differences, because it aggregates sites which are subject to different evolutionary pressures. Use of the species-specific binning method is thus likely to provide a more accurate representation of the current sequence context than the use of means, and this is what is relevant to estimates of the strength of gBGC or selection from polymorphism data. If, for example, there has been a shift toward a weaker force favoring GC in both lineages, but whose strength is nevertheless still correlated with GC content (as indicated by the analysis of *γ* below), the mean bins will on average be associated with the past GC content of the bins, so that there will be a less clear relationship of their derived variant frequencies to GC content than for the species bins. The polymorphism results for the species bins would then provide a more reliable picture than those from the mean bins.

In contrast, the mean bins are likely to provide more reliable results than the species bins when analyzing substitution patterns, since the latter are likely to introduce biases in inferences concerning the relationship between substitution patterns and GC content. Consider, for example, the bin with the lowest GC content in a given species. With a substitution rate of around 0.06 along its lineage back to its common ancestor with the other species, the expected number of changes within an SI along the lineage is of the order of 0.06 × 23 = 1.4. The chance that both of these substitutions are both *S* >*W* is thus very high. If SIs that have the lowest GC content are chosen according to the species-specific GC content, these are automatically enriched for an excess of *S *>* W* changes as opposed to *W *>* S* changes. The converse applies to SIs chosen for a high GC content. No such selection bias is expected if SIs are chosen on the basis of the mean of their GC contents for the two species.

There is another potential source of bias associated with binning, if bins with a low GC content have a higher mutational bias toward S > W mutations. This would mean that weak-strong (W/S) polymorphisms in the focal species are more likely to have S as an ancestor, accompanied by S > W mutations along an outgroup lineage, than do W/S polymorphisms in GC rich bins. This would lead to a higher probability for such bins of mis-inference of the ancestral state as W when in fact it is S. The frequency of S mutations would then be higher than expected for derived mutations for a given strength of selection, leading to an overestimation of the strength of selection or BGC in favor of S. This potential source of bias cannot, therefore, explain our inferred positive relation between *γ* and GC content. In addition, our method for estimating mutational bias, which is one of the most advanced available, shows no evidence for a relation with GC content, as we found earlier for fourfold sites (Jackson *et al.* 2017).

The biological mechanisms underlying the GC-favoring force that we have inferred are unclear. Direct experimental evidence for a GC-bias in transmission of alleles to the products of meiosis due to the repair machinery associated with recombination is limited to budding yeast, where the segregation distortion in favor of GC is modest (Mancera *et al.* 2008; but see Liu *et al.* 2018) and to mammals, where the distortion is strong at recombination hotspots (Webb *et al.* 2008; Duret and Galtier 2009; Arbeithuber *et al.* 2015). Indeed, Liu *et al.* (2018) failed to detect significantly GC-biased segregation in yeast, *Neurospora*, *Chlamydomonas* and *Arabidopsis*, although population genetics evidence has suggested its existence in yeast (Harrison and Charlesworth 2011) and *Arabidopsis* (Hämälä and Tiffin 2020). To our knowledge, there is no direct experimental evidence for gBGC, or any other form of biased transmission in *Drosophila*. In mammals, it has been hypothesized that the strength of gBGC is an adaptation to counter the high rate of mutation of methylated cytosines (Brown and Jiricny 1987; Duret and Galtier 2009). *Drosophila* has far lower levels of cytosine methylation compared to mammals (Gowher *et al.* 2000; Capuano *et al.* 2014), and the mismatch repair machinery of *Drosophila* differs from mammals and other eukaryotes in important ways (Sekelsky 2017). Consequently, it is unclear *a priori* what level of expectation there is for a GC or an AT bias in transmission of alleles in *Drosophila*. Direct observation of the progenitors and products of meiosis in *Drosophila* would be useful for testing the patterns reported here. Given that there is some doubt about the accuracy of ancestral state inference when the standard assumption of neutral evolution is applied, even using the up-to-date methods employed here, we suggest that work extending models of base composition evolution to incorporate weak directional forces (such as gBGC) would also be worthwhile (*e.g.*, Borges *et al.* 2019).

## Supplementary Material

jkab240_Supplementary_Data

## Data Availability

No new sequence data were generated in support of this research. All the code required to replicate the analyses presented here is available on Github (https://github.com/benjamincjackson/dros_gBGC). Supplementary material is available at *G3* online.
