# Supplementary material for: Evidence for a force favoring GC over AT at short intronic sites in Drosophila simulans and Drosophila melanogaster
Source: G3 (Bethesda). 2021 Jul 14;11(9):jkab240. doi: 10.1093/g3journal/jkab240 (PMC8496279; doi:10.1093/g3journal/jkab240)
Supplement: jkab240_Supplementary_Data [file jkab240_supplementary_data.docx]

Tables S1.

Base content and divergence statistics for autosomal short intron sites, binned by three different methods – 1) the mean GC content of homologous introns between *D. melanogaster* and *D. simulans* (‘mean’); 2) the species-specific GC content of introns (‘Species’), and 3) the difference in GC content between homologous *simulans* and *melanogaster* introns (‘Diff’). The statistics for each bin are: the total number of sites (num_sites), the number of substitutions from strong alleles (G or C) to weak alleles (A or T) from the ancestor of *D. melanogaster* and *D. simulans* (*ms*) to the extant *D. melanogaster* or *D. simulans* sequence (N.S.W), the number of substitutions from weak alleles to strong alleles (N.W.S), the number of neutral substitutions (N.neu), the total number of substitutions (Total changes), the GC content of *ms* (GC anc), the GC content of the extant species under consideration (GC spp) and the GC content of the species not under consideration (GC alt spp). 95% confidence intervals obtained by bootstrapping are given in parentheses.

Mean

| bin | 1 | | 2 | | 3 | | 4 | | 5 | |
| --- | --- | --- | --- | --- | --- | --- | --- | --- | --- | --- |
| species | sim | mel | sim | mel | sim | mel | sim | mel | sim | mel |
| num_sites | 30669 | 30669 | 32794 | 32794 | 33627 | 33627 | 34407 | 34407 | 35650 | 35650 |
| N.S.W. | 708.2 (634.6, 780.4) | 896.0 (812.9, 986.2) | 761.7 (689.8, 841.2) | 968.7 (886.1, 1049.1) | 789.8 (715.4, 869.6) | 995.0 (913.2, 1077.7) | 751.5 (675.6, 827.8) | 1036.9 (956.5, 1120.7) | 687.4 (629.2, 750.8) | 991.4 (914.9, 1061.8) |
| N.W.S. | 631.3 (560.7, 697.7) | 724.0 (652.0, 798.9) | 678.7 (611.2, 751.6) | 711.7 (642.8, 781.1) | 772.6 (701.4, 848.8) | 694.7 (622.8, 766.3) | 751.1 (674.8, 826.1) | 692.3 (623.6, 762.4) | 622.5 (557.6, 688.1) | 625.6 (568.8, 688.7) |
| N.neu. | 643.9 (578.8, 708.3) | 697.0 (624.9, 771.2) | 545.0 (481.5, 609.6) | 660.5 (595.8, 726.3) | 513.6 (456.8, 572.4) | 665.1 (601.3, 731.4) | 517.6 (460.6, 579.4) | 608.7 (545.1, 671.2) | 437.4 (385.8, 492.9) | 543.8 (485.3, 600.0) |
| Total.changes | 1983.4 (1857.5, 2102.2) | 2317.0 (2176.8, 2444.1) | 1985.4 (1865.1, 2124.9) | 2340.9 (2211.4, 2467.3) | 2076.0 (1960.4, 2197.2) | 2354.8 (2233.5, 2496.2) | 2020.2 (1889.2, 2149.5) | 2337.9 (2209.9, 2471.9) | 1747.3 (1638.2, 1867.5) | 2160.8 (2038.9, 2276.2) |
| GC.anc | 0.197 (0.193, 0.201) | 0.197 (0.193, 0.201) | 0.286 (0.282, 0.289) | 0.286 (0.282, 0.289) | 0.348 (0.345, 0.352) | 0.348 (0.345, 0.352) | 0.410 (0.407, 0.413) | 0.410 (0.407, 0.413) | 0.518 (0.514, 0.522) | 0.518 (0.514, 0.522) |
| GC.spp | 0.195 (0.192, 0.198) | 0.192 (0.189, 0.195) | 0.283 (0.281, 0.286) | 0.278 (0.275, 0.280) | 0.348 (0.345, 0.350) | 0.340 (0.337, 0.342) | 0.410 (0.407, 0.413) | 0.400 (0.398, 0.403) | 0.516 (0.513, 0.519) | 0.508 (0.504, 0.511) |
| GC.alt.spp | 0.192 (0.189, 0.195) | 0.195 (0.192, 0.198) | 0.278 (0.275, 0.280) | 0.283 (0.281, 0.286) | 0.340 (0.337, 0.342) | 0.348 (0.345, 0.350) | 0.400 (0.398, 0.403) | 0.410 (0.407, 0.413) | 0.508 (0.504, 0.511) | 0.516 (0.513, 0.519) |

Species

| bin | 1 | | 2 | | 3 | | 4 | | 5 | |
| --- | --- | --- | --- | --- | --- | --- | --- | --- | --- | --- |
| species | sim | mel | sim | mel | sim | mel | sim | mel | sim | mel |
| num_sites | 30665 | 30941 | 32840 | 32626 | 33660 | 33589 | 34341 | 34477 | 35641 | 35514 |
| N.S.W. | 922.3 (845.0, 1010.4) | 1161.0 (1066.2, 1253.7) | 833.8 (752.5, 910.3) | 1084.0 (997.4, 1170.4) | 748.9 (681.1, 823.6) | 951.5 (878.7, 1029.9) | 662.9 (599.6, 732.6) | 886.1 (813.2, 965.6) | 519.1 (463.4, 577.5) | 807.1 (735.3, 873.5) |
| N.W.S. | 476.4 (413.2, 537.5) | 542.5 (477.6, 601.8) | 678.5 (609.7, 750.7) | 720.0 (650.8, 791.6) | 733.7 (666.6, 808.9) | 682.8 (616.3, 754.4) | 791.3 (716.5, 865.7) | 713.1 (645.0, 783.7) | 793.6 (719.7, 869.2) | 801.4 (732.4, 867.8) |
| N.neu. | 624.4 (565.5, 692.3) | 713.7 (647.2, 786.8) | 564.5 (499.4, 634.1) | 642.7 (574.6, 714.5) | 552.7 (493.6, 609.9) | 670.1 (609.0, 732.3) | 497.4 (438.3, 558.6) | 605.2 (541.4, 665.6) | 424.6 (372.9, 479.6) | 551.1 (495.2, 611.4) |
| Total.changes | 2023.1 (1904.1, 2148.2) | 2417.2 (2273.7, 2550.2) | 2076.8 (1954.0, 2207.4) | 2446.7 (2304.8, 2583.2) | 2035.3 (1913.3, 2156.4) | 2304.4 (2185.5, 2430.3) | 1951.6 (1824.0, 2069.9) | 2204.4 (2079.0, 2326.8) | 1737.3 (1624.7, 1836.3) | 2159.6 (2038.9, 2276.4) |
| GC.anc | 0.205 (0.201, 0.209) | 0.207 (0.203, 0.212) | 0.286 (0.283, 0.290) | 0.289 (0.285, 0.293) | 0.350 (0.346, 0.353) | 0.343 (0.340, 0.347) | 0.406 (0.403, 0.410) | 0.411 (0.408, 0.414) | 0.513 (0.509, 0.517) | 0.511 (0.507, 0.515) |
| GC.spp | 0.190 (0.187, 0.193) | 0.187 (0.185, 0.190) | 0.282 (0.280, 0.284) | 0.278 (0.276, 0.280) | 0.349 (0.347, 0.351) | 0.335 (0.333, 0.337) | 0.410 (0.408, 0.412) | 0.406 (0.404, 0.408) | 0.520 (0.517, 0.524) | 0.511 (0.507, 0.514) |
| GC.alt.spp | 0.212 (0.208, 0.216) | 0.217 (0.213, 0.220) | 0.283 (0.279, 0.286) | 0.290 (0.287, 0.294) | 0.341 (0.337, 0.344) | 0.343 (0.340, 0.346) | 0.393 (0.390, 0.397) | 0.405 (0.402, 0.409) | 0.492 (0.488, 0.496) | 0.501 (0.497, 0.505) |

|  |
| --- |

Figure S1. The relationship between *D. simulans* and *D. melanogaster* GC content at the 8-30bp region of introns < 66bp long (SI sites). Open circles represent homologous introns. The correlation coefficient between the GC contents of homologous introns is 0.72 using Kendall’s rank-based measure of association.

|  |
| --- |

Figure S2. The ratios of substitution counts, plotted against the GC content of SI bins. A substitution count ratio of *N_W_* _>_ *_S_* /*N_S_* _>_ *_W_* = 1 implies equilibrium base composition. Ratios were calculated for the *D. simulans* lineage (panel A) and the *D. melanogaster* lineage (B). Here, sites were binned by species-specific GC content. Error bars represent 95% CIs from 1000 bootstraps of the data in each bin.

|  |
| --- |

Figure S3. Analyses of polymorphism data and their relationship with GC content. Top row: Derived allele frequencies (DAF) for different types of mutations. DAF was calculated for the *D. simulans* MD population (panel A) and the *D. melanogaster* ZI population (B). Middle row: Estimates of the strength of selection in favor of GC alleles ($\gamma=4N_{e}s$). Open symbols indicate values which are not significantly different from zero. Circular symbols denote models incorporating parameters that correct for polarisation error. Triangular symbols denote models not incorporating parameters that correct for polarisation error. *γ* was calculated for *D. simulans* (panel C) and *D. melanogaster* (panel D). Bottom row: The estimates of the mutational bias parameter, *κ*, calculated using the method of Glémin *et al.* (2015). *κ* was estimated from the model M1 (with *γ* ≠ 0 and polarisation errors not corrected for), for both *D. simulans* (panel E) and the *D. melanogaster* (F). Here, sites were binned by the mean GC contents of homologous introns across *D. melanogaster* and *D. simulans*. Error bars represent 95% CIs from 1000 bootstraps of the data in each bin.

|  |
| --- |

Figure S4. The ratio of substitution rates, *R* = *r_S_*_>_*_W_*/r*_W_*_>S_ plotted against the GC content of SI bins. $R$ was calculated for the *D. simulans* lineage (panel A) and the *D. melanogaster* lineage (B). Here, sites were binned by species-specific GC content. Error bars represent 95% CIs from 1000 bootstraps of the data in each bin.

|  |  |
| --- | --- |
|  |  |

Figure S5. Substitution rates for different classes of mutation. Orange triangles represent changes from strong (GC) alleles to weak (AT alleles), Green circles represent changes from weak to strong alleles. Rates were calculated for the *D. simulans* lineage (left-hand column) and the *D. melanogaster* lineage (right-hand column). Sites were binned by the mean GC content of homologous introns across *D. melanogaster* and *D. simulans* (top row) or by species-specific GC content (bottom row). Error bars represent 95% CIs derived from 1000 bootstraps of the data in each bin.

|  |  |
| --- | --- |
|  |  |

Figure S6. Median alignment scores from the MULTI-Z three-way alignment of the *D. simulans*, *D. melanogaster* and *D. yakuba* reference sequences. We extracted the alignment scores for every alignment block that contained a short intron for each bin. Error bars represent 95% confidence intervals about the median from 1000 bootstraps.

|  |  |
| --- | --- |
|  |  |
|  |  |

Figure S7. The effect of removing polymorphic sites in either species on patterns of divergence between species. Sites were binned by the mean GC contents of homologous introns in *D. simulans* (left-hand column) and *D. melanogaster* (right-hand column). Top row – the substitution rate per site for W > S substitutions (teal circles; $r_{W\to S}$) and S>W substitutions (orange triangles; $r_{S\to W}$). Middle row – the ratio of substitution rates, $R=\frac{r_{S\to W}}{r_{W\to S}}$. Bottom row – the ratio of substitution counts N_W > S_ /N_S > W_.

|  |
| --- |

Figure S8. The relationship between GC content and recombination rate. Individual short introns are represented by black circles. Red lines represent regressions with GC content as the response vector and the measure of recombination rate as the single linear predictor. For the left-hand plots, GC content is the *D. melanogaster* species-specific GC content. For the right-hand plots, GC content is the mean GC content of homologous *D. melanogaster* and *D. simulans* short introns. Three measures of recombination rate are represented: Top row, the raw recombination rates from Comeron et al. (2012); Middle row, loess-smoothed recombination rates from Comeron et al. (2012), as in Becher et al. (2020); Bottom row, using the Recombination Rate Calculator (Fiston-Lavier et al. 2010).

*References*

Becher H, Jackson BC, Charlesworth B. 2020. Patterns of genetic variability in genomic regions with low rates of recombination. Curr Biol 30:94-100 e103.

Comeron JM, Ratnappan R, Bailin S. 2012. The many landscapes of recombination in drosophila melanogaster. PLOS Genetics 8:e1002905-e1002905.

Fiston-Lavier A-S, Singh ND, Lipatov M, Petrov DA. 2010. Drosophila melanogaster recombination rate calculator. Gene 463:18-20.
